# The Global Incidence, Mortality, and Burden of Breast Cancer in 2019: Correlation With Smoking, Drinking, and Drug Use

**DOI:** 10.3389/fonc.2022.921015

**Published:** 2022-07-27

**Authors:** Leila Allahqoli, Afrooz Mazidimoradi, Zohre Momenimovahed, Azam Rahmani, Sevil Hakimi, Azita Tiznobaik, Maryam Gharacheh, Hamid Salehiniya, Farah Babaey, Ibrahim Alkatout

**Affiliations:** ^1^Midwifery Department, Ministry of Health and Medical Education, Tehran, Iran; ^2^Student Research Committee, Shiraz University of Medical Sciences, Shiraz, Iran; ^3^Department of Midwifery and Reproductive Health, Faculty of Nursing and Midwifery, Qom University of Medical Sciences, Qom, Iran; ^4^Nursing and Midwifery Care Research Center, School of Nursing and Midwifery, Tehran University of Medical Sciences, Tehran, Iran; ^5^School of Nursing and Midwifery, Tabriz University of Medical Sciences, Tabriz, Iran; ^6^Department of Midwifery and Reproductive Health, Mother and Child Care Research Center, School of Nursing and Midwifery, Hamadan University of Medical Sciences, Hamadan, Iran; ^7^Nursing Care Research Center, School of Nursing and Midwifery, Iran University of Medical Sciences, Tehran, Iran; ^8^Social Determinants of Health Research Center, Birjand University of Medical Sciences, Birjand, Iran; ^9^Head of Midwifery Department at Ministry of Health and Medical Education, Tehran, Iran; ^10^University Hospitals Schleswig-Holstein, Kiel School of Gynaecological Endoscopy, Kiel, Germany

**Keywords:** global, incidence, mortality, burden, breast cancer, smoking, drinking, drug use

## Abstract

**Background:**

Female breast cancer (FBC) is the most common type of cancer and is associated with a considerable disease burden as well as significant mortality rates. The present study aimed to provide an update on the incidence, mortality, and burden of FBC in 2019, based on the Global Burden of Disease (GBD) Study.

**Materials:**

The incidence, death rate, disability-adjusted life years (DALYs), years of life lost (YLLs), years lived with disability (YLDs), the age-standardized rates (ASR) of FBC in 204 countries, and a variety of classifications, were retrieved from the Global Burden of Disease Study. Data on tobacco use, alcohol consumption, and drug use were collected. The incidence, mortality, and burden of FBC were registered and compared between regions. Associations between age-standardized incidence rates and age-standardized mortality rates of FBC with smoking, drinking, and drug use were determined.

**Results:**

The highest incidence of FBC was observed in countries with a high socioeconomic status such as those of the European continent. Despite the lower incidence of FBC in countries with a low socio-demographic index (SDI), mortality rates secondary to FBC are higher in these countries than in high-income countries. The highest age-standardized mortality rate has been reported in the Eastern Mediterranean Region (EMRO), followed by the African Region (AFRO). The highest age-standardized rates of DALY and YLL per 100,000 population in 2019 were observed in lower-income countries, while the highest ASR of YLD per 100,000 population was reported in high-income countries.

**Conclusion:**

The present GBD-based study provides a comprehensive review of the incidence, mortality, and burden of FBC in 2019. The incidence of FBC is higher in regions with a higher socioeconomic status, whereas mortality rates and DALYs are higher in poorly developed regions. We suggest better screening measures and early detection programs for the latter regions.

## Introduction

Female breast cancer (FBC) is one of the most commonly diagnosed cancer types, with an estimated 2.3 million new cases (11.7%), and 685,000 deaths in 2020 (1). The figures are expected to reach 4.4 million in 2070 (2).

Incidence and mortality rates for FBC vary between countries. The age-standardized incidence ranges from 112.3 per 100,000 population in Belgium to 35.8 per 100,000 population in Iran. The highest age-standardized mortality rate was registered in Fiji (41.0 per 100,000 population), and the lowest in South Korea (6.4 per 100,000 population) (3).

Population structure, lifestyle, genetic factors, the environment (4), smoking, and alcohol consumption have been implicated as risk factors or protective factors for cancer (5–7). However, some studies reported the absence of a significant association between smoking and breast cancer or revealed that moderate or high consumption of alcohol did not increase the risk of breast cancer (8).

Currently, the burden of non-communicable diseases is growing due to prolonged life expectancy and lifestyle changes (9). At the end of 2020, one study reported 7.8 million living women diagnosed with breast cancer in the past 5 years (10).

Awareness of the incidence of disease and its geographic distribution is essential for effective health planning (11). In fact, the successful prevention and control of cancer depend on evidence-based policies that take epidemiological settings and the distribution of associated risk factors into account. The purpose of the present study was to provide an update on the incidence, mortality, and burden of FBC throughout the world. This is the first study to present breast cancer data based on different classifications for better understanding and interpretation. The incorporation of a variety of classifications (socio-demographic index SDI, World Health Organization WHO regions, continents, World Bank regions, and Global Burden of Disease GBD regions) permitted a comprehensive interpretation of data. These epidemiological data could also be used to devise measures of cancer prevention and screening strategies in the future.

## Materials and Methods

The aims of the present study were the following:

- To determine the incidence, mortality, and burden of FBC;
- To compare the incidence, mortality, and burden of FBC based on different classifications;
- To determine associations between age-standardized incidence rates and age-standardized mortality rates of FBC with smoking, drinking, and drug use.

FBC was defined according to the International Classification of Diseases (ICD)-10 code as C50-C50.9, D05-D05.9, D24-D24.9, D48.6, and D49.3, as well as according to the ICD-9 code as 174–175.9, 217–217.8, 233.0, 238.3, 239.3, and 610–610.9 (12).

### Data Sources

The incidence, mortality, and burden of FBC in 2019 were derived from the Global Health Data Exchange (GHDx) database for all countries in 2019, as this is the calendar year for which the most recent incidence and mortality figures are available. These data can be viewed at http://ghdx.healthdata.org. The incidence, deaths, disability-adjusted life years (DALYs), years of life lost (YLLs), years lived with disability (YLDs), and age-standardized rates of FBC were extracted from the online global burden of disease (GBD) data. The GBD has estimated epidemiological indicators of 369 diseases and injuries for both sexes in 204 countries and territories based on various divisions of countries. For an accurate interpretation, we extracted FBC data for 204 countries and for a variety of classifications based on the socio-demographic index (SDI), World Health Organization (WHO) regions, continents, World Bank regions, and GBD regions (13). The SDI is a summary measure that identifies the position of countries or geographic areas on a scale of development from 0 (lowest) to 100 (highest). The SDI is based on three factors: i) per capita income; ii) average years of schooling; and iii) total fertility rate (TFR) (14). The World Bank classifies economies for analytical purposes into four income groups: low, lower-middle, upper-middle, and high income. For this purpose, it employs gross national income (GNI) per capita in U.S. dollars, converted from the local currency based on the World Bank Atlas method. The latter is commonly used for smoothing exchange rate fluctuations (15).

The study was approved by the ethics committee of the Birjand University of Medical Sciences (ethics committee approval code IR.BUMS.REC.1400.316). As we used routinely collected anonymized electronic data, patient consent was not required. All procedures were performed in accordance with the relevant guidelines and regulations, and no personal information was disclosed or published.

### Statistical Analysis

Data were expressed as values with a 95% confidence interval (CI). Incidence, deaths, DALYs, YLLs, YLDs, and age-standardized rates were expressed as numbers per 100,000 population. Selected indicators were described separately for the individual classifications. Using the SPSS software (version 16) and Pearson’s correlation coefficient, we determined correlations between smoking, alcohol, drug use, secondhand smoke, and age-standardized incidence rates. Furthermore, we determined correlations between smoking, alcohol, drug use, secondhand smoke, and age-standardized mortality rates. To eliminate the influence of different ages in the patient population and ensure the comparability of statistical indicators, we used age-standardized rates of breast cancer incidence, death, and DALY (per 100,000 population). Definitions of the terms used are available at https://www.healthdata.org/terms-defined and https://www.healthdata.org/gbd/. A P-value less than 0.05 was considered statistically significant.

## Results

### Global Incidence of FBC

A total of 1,977,212 (95% CI; 1807615-2145215) new cases of FBC were reported worldwide in 2019, with an age-standardized incidence rate (ASR) of 45.86 per 100,000 population. The highest ASR of FBC has been reported in countries with a high SDI, such as the European region (EURO) and the American region (AMRO) (78.70-79.22 per 100,000). The lowest ASR of FBC has been reported in less developed countries such as those of the African region (AFRO) (30.99 per 100,000) (**Table 1**).

**Table 1:** Breast cancer incidence cases, age-standardized incidence rate, deaths, age-standardized mortality rates, DALYs, age-standardized DALY rates, YLLs, age-standardized YLLs rates, YLDs, and age-standardized YLDs rates in 2019.

	Incidence Cases	Incidence ASR per 105	Death Cases	Deaths ASR per 105	DALYs Cases	DALYs ASR per 105	YLLs Cases	YLLs ASR per 105	YLDs Cases	YLDs ASR per 105
	(95% CI)	(95% CI)	(95% CI)	(95% CI)	(95% CI)	(95% CI)	(95% CI)	(95% CI)	(95% CI)	(95% CI)
Global	1977212	45.86	688562	15.88	20310187	473.83	18943447.45	442.14	1366740	31.69
	(1807615-2145215)	(41.91-49.76)	(635323-739571)	(14.66-17.07)	(18744799-21866646)	(437.3-510.51)	(17533330-20455079)	(409.03-477.52)	(956851-1845097)	(22.17-42.81)
**SDI**										
High SDI	673148	79.22	165968	16.71	4046488	487.45	3536857.385	427.90	509631	59.55
	(601265-747674)	(70.83-87.7)	(150337-175159)	(15.56-17.45)	(3779696-4315580)	(459.76-518.84)	(3339275-3678535)	(409.29-443.9)	(354158-687426)	(40.9-80.6)
High-middle SDI	510299	48.93	163520	14.93	4507165	434.96	4150728.972	400.81	356436	34.15
	(458379-567966)	(43.84-54.49)	(150455-177185)	(13.75-16.19)	(4147354-4888828)	(400.69-473.31)	(3829777-4520391)	(369.56-436.91)	(245880-484148)	(23.54-46.55)
Middle SDI	485834	35.52	181116	13.66	5823380	422.91	5506431.167	399.79	316949	23.12
	(430215-545187)	(31.47-39.81)	(162719-201671)	(12.3-15.18)	(5223617-6455655)	(378.62-468.87)	(4944792-6125478)	(359.12-444.89)	(221711-433539)	(16.2-31.62)
Low-middle SDI	227241	29.47	124911	16.86	4128622	523.52	3991633.456	505.91	136988	17.61
	(199107-256008)	(25.91-33.2)	(107972-142594)	(14.59-19.24)	(3554632-4720565)	(452.09-597.21)	(3433361-4576866)	(436.79-579.05)	(98261-185102)	(12.68-23.66)
Low SDI	79445	25.67	52546	18.34	1789099	544.03	1743171.947	529.58	45927	14.45
	(69198-90893)	(22.54-29.1)	(45728-60015)	(15.98-20.84)	(1554122-2051715)	(475.61-621.56)	(1510816-2000181)	(461.76-607.36)	(32241-62245)	(10.26-19.34)
**World Bank Income Level**										
World Bank High Income	800199	78.70	205992	17.16	4951694	497.61	4345930	438.42	605764	59.19
	(712146-884095)	(70.44-87.05)	(186971-217433)	(16-17.95)	(4625980-5286767)	(470.16-529.5)	(4105307-4526338)	(419.8-455.76)	(423730-820598)	(40.94-80.24)
World Bank Upper Middle Income	695660	39.16	215208	12.08	6428678	362.61	5962318	336.36	466360	26.25
	(607551-798468)	(34.19-44.98)	(193510-240207)	(10.86-13.47)	(5759418-7159827)	(325-403.52)	(5334693-6679379)	(301.36-376.42)	(320268-639181)	(18.02-35.93)
World Bank Low Income	48405	25.31	32242	18.17	1067534	524.78	1039563	510.52	27971	14.26
	(40084-58453)	(21.11-30.38)	(27036-38568)	(15.37-21.58)	(884804-1293150)	(438.59-632.52)	(863347-1257149)	(426.97-614.17)	(19232-38245)	(9.92-19.28)
World Bank Lower Middle Income	431691	31.71	234614	18.13	7846711	562.49	7580882	543.15	265828	19.35
	(383142-481139)	(28.16-35.32)	(206213-263527)	(15.9-20.3)	(6873144-8793109)	(493.2-629.13)	(6622986-8515481)	(475.55-609.7)	(186710-357133)	(13.65-25.91)
Continents										
Africa	117925	30.99	69432	20.06	2294711	570.77	2223889	552.63	70822	18.14
	(101730-134951)	(26.98-35.24)	(60108-79920)	(17.47-22.9)	(1955441-2668404)	(491.94-657.91)	(1895557-2581266)	(476.09-638.58)	(48919-96239)	(12.74-24.46)
America	429443	65.20	117666	17.16	3201110	496.90	2899437	451.22	301673	45.68
	(374960-489456)	(57.06-74.34)	(109358-124410)	(16.1-18.12)	(3015772-3408746)	(467.77-528.93)	(2746515-3061600)	(427.48-476.88)	(210576-411683)	(31.88-62.45)
Asia	914878	35.88	337822	13.41	10889308	425.95	10274340	401.82	614968	24.13
	(815789-1025502)	(32.01-40.17)	(301545-375251)	(11.99-14.89)	(9751379-12107339)	(381.43-474.1)	(9161299-11492533)	(358.74-449.09)	(424712-840118)	(16.64-32.97)
Europe	511346	69.77	162459	19.08	3892322	539.11	3515331	487.92	376991	51.19
	(455963-572867)	(62.44-78.4)	(147510-173152)	(17.63-20.29)	(3617767-4184068)	(503.03-579.15)	(3288370-3733598)	(459.92-519.72)	(262504-517829)	(35.24-70.64)
**WHO Regions**										
African Region	89504	30.03	56906	20.91	1850849	581.45	1798449	564.39	52401	17.05
	(76303-104339)	(25.85-34.59)	(47929-66423)	(17.87-24.06)	(1537100-2189049)	(487.56-680.12)	(1492978-2124462)	(472.87-663.26)	(36154-71996)	(11.94-23.12)
Region of the Americas	429443	65.20	117666	17.16	3201110	496.90	2899437	451.22	301673	45.68
	(374960-489456)	(57.06-74.34)	(109358-124410)	(16.1-18.12)	(3015772-3408746)	(467.77-528.93)	(2746515-3061600)	(427.48-476.88)	(210576-411683)	(31.88-62.45)
South-East Asia Region	250266	26.03	135751	14.70	4512018	460.98	4357350	445.02	154668	15.97
	(211148-292317)	(22.01-30.41)	(113674-160384)	(12.33-17.35)	(3779387-5324973)	(386.42-543.77)	(3636515-5165128)	(372.13-527.55)	(107140-215120)	(11.1-22.23)
European Region	527648	68.30	168748	19.06	4092582	539.08	3704377	489.05	388205	50.03
	(470623-590560)	(61.17-76.76)	(153465-179630)	(17.63-20.33)	(3809617-4386078)	(504.6-578.22)	(3471969-3941794)	(461.19-520.84)	(270802-533993)	(34.56-69.09)
Eastern Mediterranean Region	121096	47.56	58460	25.20	2067425	780.99	1991787	751.67	75639	29.32
	(104300-142149)	(41.04-55.56)	(49389-70621)	(21.43-30.16)	(1736241-2504651)	(659.45-936.68)	(1661070-2417053)	(632-909.18)	(53062-104208)	(20.72-40.22)
Western Pacific Region	551442	40.21	148704	10.59	4519164	331.65	4130152	303.29	389012	28.36
	(463836-646269)	(33.74-47.29)	(128533-170771)	(9.14-12.16)	(3927197-5172192)	(288.07-379.58)	(3555789-4776147)	(260.91-351.02)	(260749-544120)	(19-39.65)
**GBD Region**										
**East Asia & Pacific - WB**	635424	39.61	189458	11.67	5949139	374.63	5505247	346.96	443892	27.67
	(543840-737370)	(34-45.99)	(167254-214308)	(10.32-13.2)	(5266421-6720579)	(331.57-423.38)	(4834411-6260908)	(304.18-394.1)	(299978-614547)	(18.69-38.27)
Southeast Asia	138540	38.52	66463	19.23	2264886	621.22	2175392	596.44	89493	24.78
	(118944-161237)	(33.11-44.64)	(57087-76424)	(16.62-22.01)	(1940149-2631321)	(534.07-719.1)	(1857361-2534943)	(509.27-692.2)	(62118-126345)	(17.28-34.74)
Oceania	2990	65.58	1800	42.80	68056	1416.87	66268	1378.32	1788	38.55
	(2286-3842)	(50.44-83.58)	(1377-2288)	(33.19-54.23)	(51630-87902)	(1084.79-1808.02)	(50257-85760)	(1054.75-1756.46)	(1175-2549)	(25.58-54.16)
East Asia	382321	35.69	98162	9.12	3024987	282.15	2761185	257.51	263802	24.64
	(303308-477173)	(28.32-44.54)	(79216-120112)	(7.36-11.13)	(2477984-3659370)	(230.81-341.19)	(2215670-3402444)	(206.02-317.51)	(172343-374748)	(16.07-34.95)
Sub-Saharan Africa - WB	86379	29.44	56397	21.01	1836514	583.59	1786394	567.05	50120	16.54
	(73271-100548)	(25.31-33.82)	(47548-65712)	(18.03-24.14)	(1532349-2158987)	(489.35-680.64)	(1486586-2109202)	(475.46-661.74)	(34469-68558)	(11.61-22.26)
Central Sub-Saharan Africa	9708	28.98	6846	22.42	228643	627.53	223188	611.88	5455	15.65
	(6954-12820)	(20.86-38.55)	(4964-9007)	(16.16-29.76)	(165704-300184)	(452.46-827.77)	(161258-292281)	(441.3-806.97)	(3458-7980)	(9.95-22.69)
Eastern Sub-Saharan Africa	23906	24.04	16395	18.15	541257	501.54	527472	488.22	13785	13.32
	(20170-27839)	(20.78-27.49)	(14024-18922)	(15.65-20.6)	(454613-633649)	(427.72-580.64)	(442165-617176)	(416.07-564.66)	(9503-18804)	(9.34-17.82)
Southern Sub-Saharan Africa	11543	33.89	7123	22.06	206574	588.03	199785	568.45	6789	19.58
	(10256-12971)	(30.14-38.02)	(6329-7946)	(19.72-24.54)	(181578-235192)	(517.74-667.08)	(175837-227986)	(500-644.44)	(4803-9091)	(13.83-26.06)
Western Sub-Saharan Africa	37976	32.91	24513	23.25	805234	650.96	783211	632.43	22023	18.53
	(29485-46862)	(25.93-40.11)	(19227-30840)	(18.65-28.62)	(619155-1027292)	(506.41-818.74)	(602716-1003237)	(492.58-800.34)	(14504-31728)	(12.44-26.1)
**South Asia - WB**	221921	27.71	128282	16.74	4250350	517.56	4117759	501.19	132590	16.37
	(184132-262993)	(23.05-32.75)	(106022-152432)	(13.89-19.84)	(3500784-5062660)	(427.33-615.3)	(3365390-4925789)	(409.74-598.72)	(91711-184565)	(11.34-22.7)
South Asia	215790	27.72	125312	16.83	4153491	520.59	4024753	504.24	128738	16.35
	(178051-256860)	(22.91-33)	(103075-149357)	(13.91-20)	(3398962-4965685)	(426.84-620.44)	(3278008-4824565)	(411.84-604.47)	(88599-178968)	(11.23-22.68)
**Latin America & Caribbean - WB**	151700	41.68	57385	15.77	1670794	458.46	1577646	432.84	93147	25.61
	(135030-171454)	(37.1-47.11)	(52852-62403)	(14.53-17.15)	(1529066-1830428)	(419.65-502.26)	(1450766-1724564)	(398-473.11)	(66502-126815)	(18.29-34.86)
Andean Latin America	8966	29.63	3762	12.67	113072	370.35	107792	352.94	5280	17.41
	(7271-11032)	(24.05-36.45)	(3098-4614)	(10.44-15.51)	(91324-140750)	(300.19-459.51)	(86478-134069)	(283.61-438.11)	(3597-7389)	(11.88-24.36)
Caribbean	14940	55.37	5705	20.84	166491	623.62	157552	590.42	8939	33.20
	(12616-17581)	(46.63-65.11)	(4847-6668)	(17.62-24.4)	(138081-197239)	(515.19-740.86)	(130933-187179)	(489.09-702.41)	(6244-12147)	(23.16-45.17)
Tropical Latin America	53196	39.75	20299	15.19	604637	451.18	572812	427.38	31825	23.79
	(49824-56529)	(37.24-42.24)	(18912-21540)	(14.15-16.11)	(568713-642189)	(424.36-479.09)	(539848-607346)	(402.86-453.37)	(22912-42474)	(17.13-31.76)
Central Latin America	50560	38.45	16681	12.87	516955	390.08	486632	367.02	30323	23.06
	(42504-60048)	(32.3-45.64)	(14310-19573)	(11.05-15.09)	(438008-610427)	(330.64-460.41)	(410526-573055)	(310.23-431.7)	(21338-42029)	(16.26-31.9)
**Middle East & North Africa - WB**	77542	40.92	28284	16.41	983031	505.45	931471	478.30	51560	27.14
	(66602-90120)	(35.19-47.28)	(24391-32525)	(14.26-18.76)	(841640-1142388)	(434.06-583.14)	(797152-1080104)	(410.11-552.38)	(35675-70795)	(18.9-37.02)
North Africa and Middle East	94746	37.48	35405	15.22	1222835	472.73	1160511	448.19	62324	24.54
	(82334-108875)	(32.68-42.94)	(30676-40571)	(13.31-17.35)	(1053073-1411009)	(409-544.75)	(998083-1346371)	(386.27-516.64)	(43715-85058)	(17.21-33.46)
**Europe & Central Asia - WB**	521786	68.21	166894	19.03	4046335	538.26	3662455	488.31	383881	49.95
	(466049-584666)	(61.16-76.49)	(151764-177582)	(17.6-20.28)	(3766546-4331939)	(503.58-577.48)	(3432874-3897645)	(460.81-520.27)	(267909-527423)	(34.52-68.98)
Central Asia	17746	38.36	7531	17.29	246485	523.88	234678	498.38	11806	25.50
	(15766-19917)	(34.23-42.8)	(6746-8410)	(15.53-19.16)	(217860-278216)	(464.12-588.67)	(208230-263855)	(443.11-558.66)	(8354-16249)	(18.06-35.12)
Central Europe	60774	60.22	23042	19.87	555788	552.66	514426	511.59	41361	41.07
	(52615-69893)	(52.04-69.57)	(20121-26224)	(17.25-22.71)	(483445-638980)	(476.95-638.66)	(446089-588962)	(441.31-590.39)	(28994-56209)	(28.53-56.24)
Eastern Europe	93968	51.89	34965	17.47	955439	529.13	890569	493.42	64870	35.71
	(80449-110269)	(44.14-61.31)	(30283-40415)	(15.05-20.36)	(831143-1113076)	(456.07-618.57)	(763308-1042540)	(420.37-579.16)	(43899-91942)	(24.09-50.85)
**High Income**	759541	80.90	194557	17.41	4635358.945	505.10	4057221.57	443.92	578137	61.17
	(672521-843233)	(71.79-89.93)	(176309-205023)	(16.22-18.15)	(4337633-4944572)	(477.36-536.5)	(3834179-4216286)	(426.06-459.42)	(402958-785398)	(42.26-83.25)
Australasia	19150	84.69	4449	17.47	113161	509.03	98386	444.16	14775	64.87
	(15494-23737)	(68.25-104.99)	(4005-4799)	(16.11-18.69)	(104132-123470)	(471.05-552.78)	(91489-104548)	(415.78-470.75)	(9958-21138)	(43.73-93.2)
Western Europe	338607	85.85	97509	19.79	2165271	552.56	1906075	487.10	259196	65.46
	(292300-386967)	(74.12-98.85)	(87377-103327)	(18.32-20.77)	(2015070-2318468)	(519.78-591.68)	(1785491-1993233)	(463.36-507.1)	(179277-358787)	(44.7-91.4)
Southern Latin America	24595	56.51	11145	24.04	275603	643.67	258488	604.15	17115	39.52
	(19218-31147)	(43.78-71.94)	(10309-11914)	(22.41-25.61)	(257573-296679)	(602.3-691.61)	(242865-274897)	(568.83-642.26)	(11330-24255)	(25.98-56.45)
North America	280050	93.75	60931	18.36	1546697	533.81	1336738	463.96	209959	69.84
	(233456-334720)	(78.03-112.64)	(56307-64176)	(17.27-19.19)	(1448016-1652270)	(502.47-569.79)	(1276096-1391052)	(444.49-481.75)	(142244-292801)	(46.9-97.74)
Asia-Pacific	97168	56.30	20529	9.78	534726	321.94	457616	278.10	77109	43.84
	(80971-115106)	(47.14-67.18)	(17761-22336)	(8.91-10.41)	(484951-582464)	(300.1-349.17)	(419729-485389)	(262.07-293.02)	(51718-109392)	(29.19-62.67)

### Global FBC Mortality

In 2019, the number of deaths due to FBC worldwide were 688,562 (95% CI; 635,323-739,571), and the age-standardized mortality rate was 15.88 (95% CI; 14.66-17.07). Countries with a low SDI had the highest age-standardized mortality rate of 18.34, whereas countries with a high and middle SDI had age-standardized mortality rates of 16.71 and 13.66, respectively. Among the continents, the highest age-standardized mortality rate was reported in Africa and the lowest in Asia (**Table 1**).

### Global Disability-Adjusted Life Years for FBC

In 2019, the number of DALYs due to FBC worldwide were 20,310,187 (95% CI 18,744,799-21,866,646), of which 18,943,447 were related to YLLs and 1,366,740 to YLDs.

The global ASR-DALY, ASR-YLLs, and ASR-YLDs due to FBC were 473.83, 442.14, and 31.69, respectively. The highest ASR of DALYs per 100,000 population in 2019 was noted in lower middle-income countries [562.49; 95% CI (493.2-629.13)], followed by low-income [524.78; 95% CI (438.59-632.52)] countries. The lowest ASR-DALYs were registered in countries with a middle SDI. The ASR of YLLs per 100,000 population was highest in Africa [552.63; 95% CI (476.09-638.58)] and lowest in Asia [425.95; 95% CI (381.43-474.1)]. However, with regard to the ASR of YLDs per 100,000 population, the highest rate was noted in high-income countries [59.19; 95% CI (40.94-80.24)], and the lowest in low-income countries [14.26; 95% CI (9.92-19.28)] (**Table 1**).

### Correlation Between Global FBC Incidence, Mortality, and Smoking, Drinking, and Drug Use

**Figure 1:**
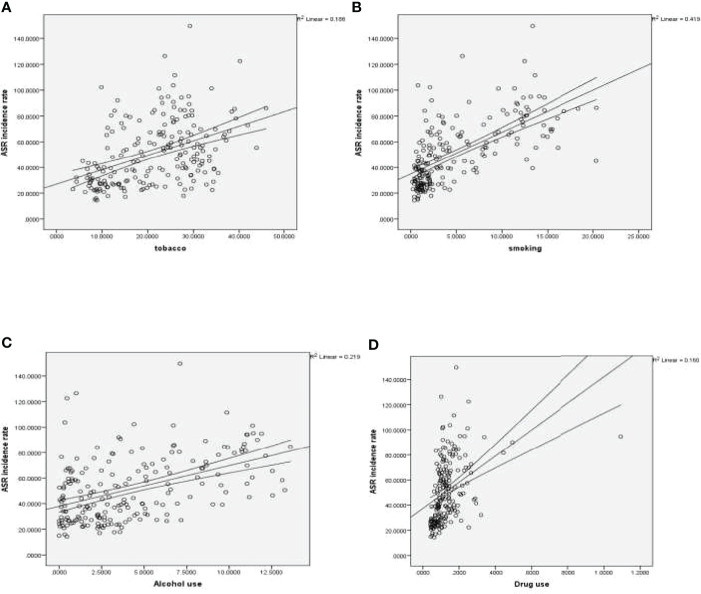
**(A)** Correlation between age-standardized incidence rate of FBC and tobacco. **(B)** Correlation between age-standardized incidence rate of FBC and smoking. **(C)** Correlation between age-standardized incidence rate of FBC and alcohol. **(D)** Correlation between age-standardized incidence rate of FBC and drug use.

The age-standardized incidence rates of FBC increased with the use of tobacco (r = 0.43, p < 0.001), smoking (r = 0.64, p < 0.001), alcohol (r = 0.48, p < 0.001), and drugs (r = 0.4, p < 0.001) (**Figures 1A–D**). No statistically significant correlation was registered between age-standardized incidence rates of FBC and secondhand smoke.

The age-standardized mortality rate for FBC was significantly correlated with secondhand smoke (r = 0.156, p = 0.25).

## Discussion

The significance of FBC is evidenced by its high incidence and mortality rates. In most countries, FBC is among the leading causes of death (16). We addressed the incidence, mortality rates, and disease burden of FBC based on a variety of classifications, as well as the association between the disease and smoking, drinking, and drug use in 2019. The present study revealed the highest incidence of FBC in countries with a high socioeconomic status such as the EURO. The higher incidence of FBC in regions with a higher socioeconomic status may be at least partly attributed to drastic changes in lifestyle and the built environment, which have influenced risk factors for breast cancer such as obesity, alcohol consumption (17), urbanization, sedentary behavior (18, 19), postponement of childbearing, lower overall fertility rates, lower breastfeeding rates (1, 20–22), and the use of hormone replacement therapy (23). Another important risk factor that might explain the difference is early detection rates and screening measures in countries with a higher socioeconomic status (20, 24). Screening investigations disclose cancer in earlier asymptomatic stages and identify cases that would not have been diagnosed otherwise. Another reason for the high incidence of breast cancer in countries with a higher socioeconomic status may be the availability of, and actual access to, treatment (21). Furthermore, the burden of cancer in countries with a lower socioeconomic status may be underestimated because these countries frequently lack reliable cancer registries and reporting systems (25). The systems of reporting epidemiological data in regions with a higher socioeconomic status are generally more robust (24, 26, 27). In view of these reporting biases (28), data concerning the incidence of cancer across regions should be interpreted with caution.

The present investigation revealed lower incidence rates of FBC in the EMRO and AFRO regions, yet higher mortality rates secondary to FBC in these regions than in countries with a higher incidence of the disease. The higher mortality rates secondary to FBC in regions with a lower socioeconomic status may be largely attributed to limited resources and the absence of well-organized health policies, leading to inadequate diagnosis, late-stage presentation, and untimely and inappropriate treatment (29–32). These reasons were confirmed in a study which reported that more than half of women with breast cancer in the Middle East are diagnosed in the third and fourth stages of the disease with lymph node involvement (33). In countries with a high socioeconomic status, on the other hand, very few women are diagnosed in the third and fourth stages of the disease (34). The fact that 69% of patients diagnosed with advanced stages die within five years after treatment (32) might explain the high mortality rates in the EMRO and AFRO regions. One of the important factors contributing to poor health in the EMRO is war, which causes large numbers of displaced persons, disruption of care structures and supplies, lack of qualified healthcare personnel, and financial restraints on patients and healthcare systems in countries with a large influx of refugees (35, 36).

While the traditional cancer metrics of incidence and mortality are crucial, DALY estimates provide a view of the healthy years of life lost due to cancer morbidity and mortality on a global basis (37). In the present study, the highest ASR of DALYs per 100,000 population was observed in the EMRO and AFRO regions, but the highest ASR of YLDs per 100,000 population was registered in Europe followed by the United States, while the lowest rate was noted in Africa. The high rates of YLDs in higher SDI settings is consistent with improved survival (38), as well as wider access to cancer screening (39, 40), diagnosis (41, 42), and treatment (43, 44) with increasing SDI. The highest ASR of YLLs were noted in low SDI regions.

The incidence and mortality rates of cancer are growing rapidly worldwide, reflecting aging, population growth, and changes in the prevalence and distribution of the principal risk factors for cancer. Many of these risk factors are associated with socioeconomic development (45). Taken together, these data emphasize the need for intensified efforts to prevent and control cancer (46), as well as the need to accelerate progress in lower SDI areas and thus reduce the impact of the growing burden (47, 48). One important step in this regard is educating women in all countries about early detection and treatment. Tailored integration of cancer into health planning may serve to reduce the global burden of the disease and eliminate the current inequities between transitioning and transitioned countries.

### Strengths and Limitations

This GBD-based study is an update of the incidence patterns, mortality, DALYs associated with FBC worldwide, and the most relevant risk factors. The limitations of the present analysis are worthy of mention. First, it is based on GBD 2019 and thus shares the overall limitations described in previous publications (49, 50), including the challenges of quantifying all sources of uncertainty, lags in data availability, variations in coding practices, and other biases. Second, the paucity of reliable cancer registries and reporting systems in countries with a low SDI may have influenced the interpretation of data. Third, the investigation did not encompass a comparative assessment of other risk factors. Data concerning other risk factors were not available in the online dataset. For the above-mentioned reasons, the current data should be interpreted with caution.

## Conclusion

This GBD-based 2019 study provides a comprehensive summary of the incidence, mortality, and burden of breast cancer, which is one of the most common types of cancer in the world. Despite the higher incidence of the disease in regions with a higher socioeconomic status, mortality rates and DALYs secondary to breast cancer are higher in poorly developed regions. The reasons for this phenomenon are poor accessibility to novel drugs and the limited application of clinical guidelines in the latter regions. Steps should be taken to reduce the burden of breast cancer, especially in lower SDI countries, and halt the acceleration of disparities. Patients in underdeveloped countries are affected to a greater extent by the burden of the disease. The information reported in this study illustrates the global disease burden of breast cancer and may be used to guide the allocation of healthcare resources for the prevention, screening, and treatment of the disease.

## Data Availability Statement

The data presented in this study are available on request from corresponding author.

## Author Contributions

LA, HS, and IA designed and conceived the study. AM and HS collected the data. LA, AM, HS, and IA analyzed and interpreted the data. LA, AM, ZM, AR, SH, MGH, and FB, drafted manuscript. HS and IA provided administrative, technical, or material support. LA and IA provided oversight. All authors contributed to the article and approved the submitted version.

## Conflict of Interest

The authors declare that the research was conducted in the absence of any commercial or financial relationships that could be construed as a potential conflict of interest.

## Publisher’s Note

All claims expressed in this article are solely those of the authors and do not necessarily represent those of their affiliated organizations, or those of the publisher, the editors and the reviewers. Any product that may be evaluated in this article, or claim that may be made by its manufacturer, is not guaranteed or endorsed by the publisher.
